# Which Explanatory Variables Contribute to the Classification of Good Visual Acuity over Time in Patients with Branch Retinal Vein Occlusion with Macular Edema Using Machine Learning?

**DOI:** 10.3390/jcm11133903

**Published:** 2022-07-04

**Authors:** Yoshitsugu Matsui, Kazuya Imamura, Shinichiro Chujo, Yoko Mase, Hisashi Matsubara, Masahiko Sugimoto, Hiroharu Kawanaka, Mineo Kondo

**Affiliations:** 1 Department of Ophthalmology, Mie University Graduate School of Medicine, 2-174, Edobashi, Tsu 514-8507, Mie, Japan; shin-mack10@hotmail.co.jp (S.C.); yokosun9@gmail.com (Y.M.); hmatsu@med.mie-u.ac.jp (H.M.); sugmochi@clin.medic.mie-u.ac.jp (M.S.); mineo@clin.medic.mie-u.ac.jp (M.K.); 2 Department of Electrical and Electronic Engineering, Mie University, 1577, Kurimamachiyacho, Tsu 514-8507, Mie, Japan; 417311@m.mie-u.ac.jp (K.I.); kawanaka@elec.mie-u.ac.jp (H.K.)

**Keywords:** optical coherence tomography, branch retinal vein occlusion, fovea, macular, logistic regression, machine learning, clinical prediction models

## Abstract

This study’s goal is to determine the accuracy of a linear classifier that predicts the prognosis of patients with macular edema (ME) due to a branch retinal vein occlusion during the maintenance phase of antivascular endothelial growth factor (anti-VEGF) therapy. The classifier was created using the clinical information and optical coherence tomographic (OCT) findings obtained up to the time of the first resolution of ME. In total, 66 eyes of 66 patients received an initial intravitreal injection of anti-VEGF followed by repeated injections with the pro re nata (PRN) regimen for 12 months. The patients were divided into two groups: those with and those without good vision during the PRN phase. The mean AUC of the classifier was 0.93, and the coefficients of the explanatory variables were: best-corrected visual acuity (BCVA) at baseline was 0.66, BCVA at first resolution of ME was 0.51, age was 0.21, the average brightness of the ellipsoid zone (EZ) was −0.12, the intactness of the external limiting membrane (ELM) was −0.14, the average brightness of the ELM was −0.17, the brightness value of EZ was −0.17, the area of the outer segments of the photoreceptors was −0.20, and the intactness of the EZ was −0.24. This algorithm predicted the prognosis over time for individual patients during the PRN phase.

## 1. Introduction

Branch retinal vein occlusion (BRVO) is a common form of retinal vascular disease [1]. The pathogenesis of BRVO is multifactorial with contributions from mechanical obstruction, degeneration of the vessel wall, and hematologic abnormalities such as inflammatory disorders and thrombophilia in at-risk individuals. The resulting venous obstruction leads to the elevation of venous pressure upstream of the crossing that may overload the collateral drainage capacity, resulting in intraretinal hemorrhages, macular edema, and ischemia [2]. Macular edema (ME) associated with branch retinal vein occlusion (BRVO) causes a rapid reduction in best-corrected visual acuity (BCVA) [3]. Repeated intravitreal injections of antivascular endothelial growth factor (anti-VEGF) can improve BCVA in most cases after 1 year [4]. The number of injections in the first year ranged from 2 to 4.9 with the initial anti-VEGF injection followed by repeated injections with the pro re nata (PRN) regimen [5,6,7]. These findings indicate that ME with acute BCVA reduction recurred 1 to 3.9 times after the first anti-VEGF injection in that study. During the first year of treatment, clinicians need to consider the variations in disease process and BCVA to be able to determine the long-term visual acuity. For this reason, one of the most important types of data that patients want to know is the clinical prognosis of individual patients if they continue treatment. One way to solve this problem is to use machine-learning prediction algorithms. Clinical predictions are performed on the basis of individual patient variables. Thus, Gallardo and colleagues reported that it was possible to predict the treatment demand in real-world BRVO patients using machine learning from a small dataset [8]. Regarding visual function prognosis, many factors that were already known were significantly correlated with the final BCVA [9,10,11,12,13,14,15]. However, the causal relationships and inter-relationships are complex, and it is difficult for clinicians to predict visual function after the continuous treatment of an individual patient at an early point of the treatment. During the first-year maintenance phase of anti-VEGF therapy, our group has shown that a nonlinear algorithm could predict BCVA in patients with ME due to BRVO. This was achieved by using the clinical information and optical coherence tomographic (OCT) findings obtained at the time of the first resolution of the ME after the initial treatment [16]. However, the degree of the contribution of each explanatory variable to the classification was not determined.

Thus, the purpose of this study was to determine the degree of contribution of each explanatory variable to the classification. To accomplish this, we set up a single-label two-class problem to classify the patients into two groups: Group A with good visual outcome, and Group B with poor visual outcome. We determined the accuracy of the new linear classifier and the degree of contribution of the explanatory variables.

## 2. Materials and Methods

### 2.1. Patients and Treatment Regimen

This was a single-center, retrospective cohort study of the medical records of patients examined in the Department of Ophthalmology, Mie University Hospital from May 2011 to March 2021.

The participants of this study were the same ones as those in our previous study [16]. Patients with acute BRVO with ME who had received one initial anti-VEGF injection followed by a monthly PRN regimen with no loading phase and 12 months of follow-up examinations were studied. The eligibility criteria were: BRVO was the only disease causing the visual acuity reduction; no prior treatment for ME such as anti-VEGF treatments, laser photocoagulation, intraocular surgery, or intravitreal steroid injection, before the initiation of this study and during the follow-up period; the ME was resolved at 1 to 2 months after the first anti-VEGF treatment; cataract surgery had not been performed during the postoperative period; and cases in which there were no missing values in the explanatory variables. None of the patients had bilateral BRVO.

Each patient received one initial intravitreal injection of anti-VEGF agent followed by a monthly PRN regimen (1 + PRN). At the monthly visits, a repeat injection was given if the mean central foveal thickness (CFT) was >300 µm or if subretinal fluid (SRF) was present at the fovea in the OCT images. Spectral domain (SD) OCT images were recorded with the Spectralis OCT (Spectralis HRA + OCT, Heidelberg Engineering, Heidelberg, Germany) at the baseline and every month after the initial anti-VEGF injection.

### 2.2. Grouping into Two Groups Based on Visual Acuity over Time

The BCVA was measured at the baseline and at every visit after the initial anti-VEGF treatment. We focused on the BCVA over time, and defined Group A patients as those with good prognosis whose maximal BCVA was ≤0 logarithm of the minimal angle of resolution (logMAR) units and a minimal BCVA of ≤0.15 logMAR units after the first resolution of the ME for up to 12 months after the first treatment. Group B consisted of all other cases. One of the cutoff values for BCVA, 0.15 logMAR units, was adopted because it is the standard value for visual acuity required for renewal of a Japanese driver’s license.

### 2.3. Preprocessing of OCT Images and Features from OCT Images

**Figure 1:**
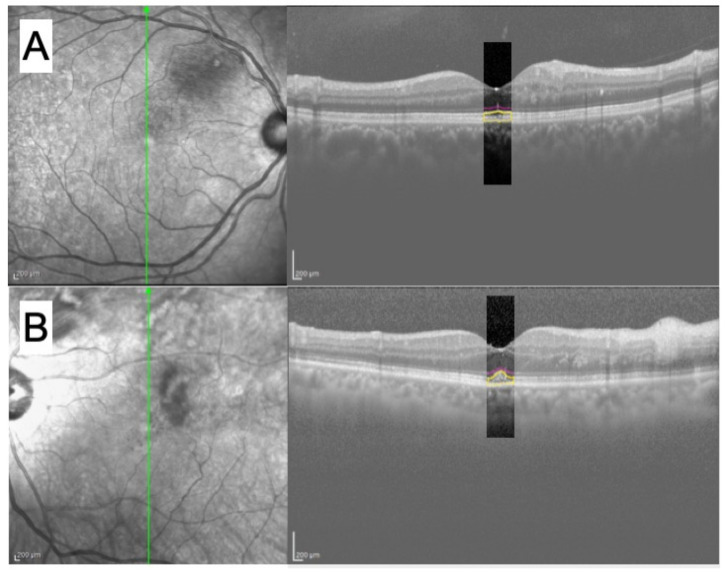
(**A**,**B**) SLO and OCT images of a representative case of branch retinal vein occlusion (BRVO) classified as Groups A and B, respectively, at the time of ME resolution. Each green line represents a scanned line in the fundus. OCT image on the right is a trimmed image of the original image. In the trimmed image, the ELM (pink) and EZ (yellow) lines are annotated, and the area surrounded by the EZ and RPE (yellow) lines. ELM = external limiting membrane; EZ = elliptical zone; RPE = retinal pigment epithelium.

The B-scan vertical tomographic images taken just prior to the first resolution of the ME were used to create some of the handcrafted features. The original size of the OCT image was 768 × 496 pixels (px; 9 mm× 1.9 mm, 30°), and it was cropped to create a 50 × 256 px (0.59 mm × 0.98 mm, 1.96°) trimmed image. The trimming process was manually performed with the foveal bulge or foveal pit as the center of trimmed image. This was conducted by one of the authors (YM). From the trimmed images, the features related to the visual acuity were defined as the handcrafted features as performed in earlier studies [11,12,13,14]. Annotation was added to the trimmed images with the following method. First, the ELM, EZ, and RPE were manually marked in the trimmed images by an examiner (YM) using image-editing software GIMP [17]. The ELM, EZ, and RPE lines were drawn (Figure 1).

Then, 5 types of handcrafted features were created for the objective evaluations. The handcrafted features were: (1) ELM continuity-org, the sum of the brightness values under the ELM line; (2) EZ continuity-org, the sum of the brightness values under the EZ line; (3) ELM clarity, the sum of the ratio between the brightness value below the ELM line and the average brightness values of 3 pixels above and below it; (4) EZ clarity, the total sum of the ratio between the brightness value below the EZ line and the average value of the brightness values of 3 pixels above and below it; and (5) Area was the area surrounded by the EZ and RPE lines. These handcraft features were set as continuous quantity variables. The brightness values of OCT images were in the range from 0 to 255, and the brightness of the ELM and EZ lines was determined with the following equation for standardization; the optimized brightness of ELM or EZ line = 255/(255 − B). The brightness of the ELM or EZ line was defined as A, and the brightness of the liquefied vitreous body was defined as B. This method of standardizing the image brightness was based on our previous paper [18]. For Factors 1–4, when there was no line, the brightness value of that pixel was added as 1.

We also created two handcrafted features on the basis of the subjective evaluations of the trimmed images. The continuity of each EZ and ELM line was subjectively classified to three levels: vanishing, discontinuous, and continuous, and each was assigned a value of 0, 1, or 2, respectively. These handcrafted features were set as categorical variables.

### 2.4. Selection of Explanatory Variables

**Figure 2:**
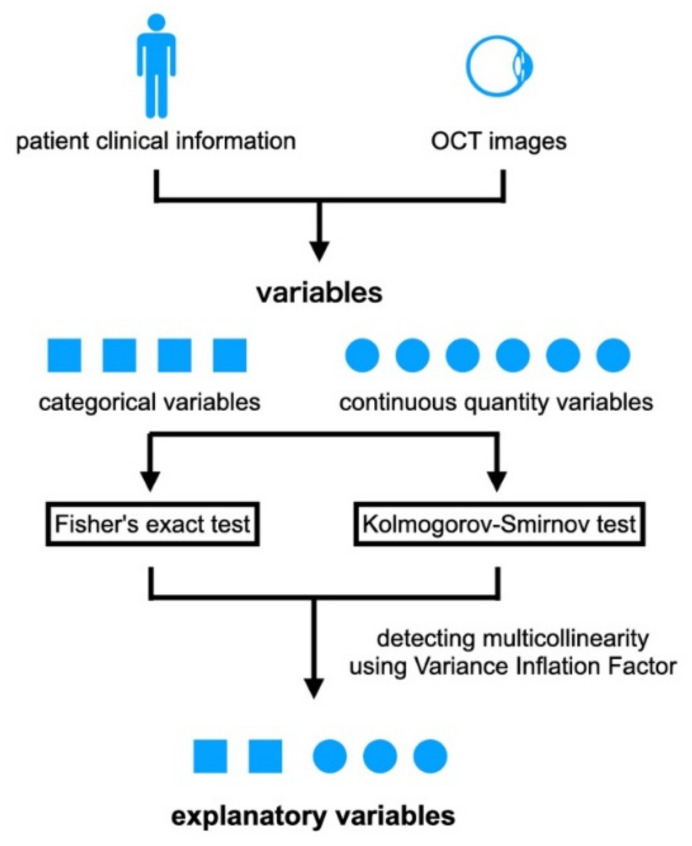
Fisher’s probability of correct answer test was conducted for categorical variables for variables extracted from clinical information and OCT images. K–S tests were conducted for continuous quantity variables. Variables that had a statistically significant difference between Groups A and B were used as candidates for explanatory variables. Among these variables, those with variance inflation factor values greater than 10, that is, those with R^2^ scores greater than 0.9, were eliminated. One variable with the highest R^2^ score greater than 0.9 was eliminated, and the tests were conducted until the R^2^ score for all variables was less than 0.9. Lastly, the remaining variables were used as the explanatory variables.

Variables that were statistically significantly different between the two groups were used as candidates for the explanatory variables (Figure 2). Comparisons of the variables and handcraft features between the two groups were performed using the following tests. The Kolmogorov–Smirnov test was used for the continuous variables, i.e., the age, interval from onset to treatment, BCVA (logMAR units) at the baseline, interval between initial treatment to the first resolution of the ME, BCVA at the time of first resolution of the ME, ELM-continuity-org, ELM-clarity, EZ-continuity-org, EZ- clarity, and Area. Fisher’s exact tests were used to determine the significance of the differences in the categorical variables of the sex, disease type, left or right affected eye, the location of lesions, and the integrity of the ELM and EZ. The Wilcoxon test was used to determine the significance of the association between the BCVA at the baseline and at 12 months. The findings were taken to be statistically significant when *p* < 0.05. Then, among those variables with variance inflation factor values that were greater >10, i.e., R^2^ scores greater than 0.9, were eliminated. One variable with the highest R^2^ score greater than 0.9 was also eliminated, and the test was conducted until the R^2^ score for all variables was less than 0.9. Lastly, the remaining variables were used as the explanatory variables. All statistical analyses were performed using statistical programming language R (R version 3.1.3; e foundation for Statistical Computing, Vienna, Austria).

### 2.5. Machine-Learning Algorithm

**Figure 3:**
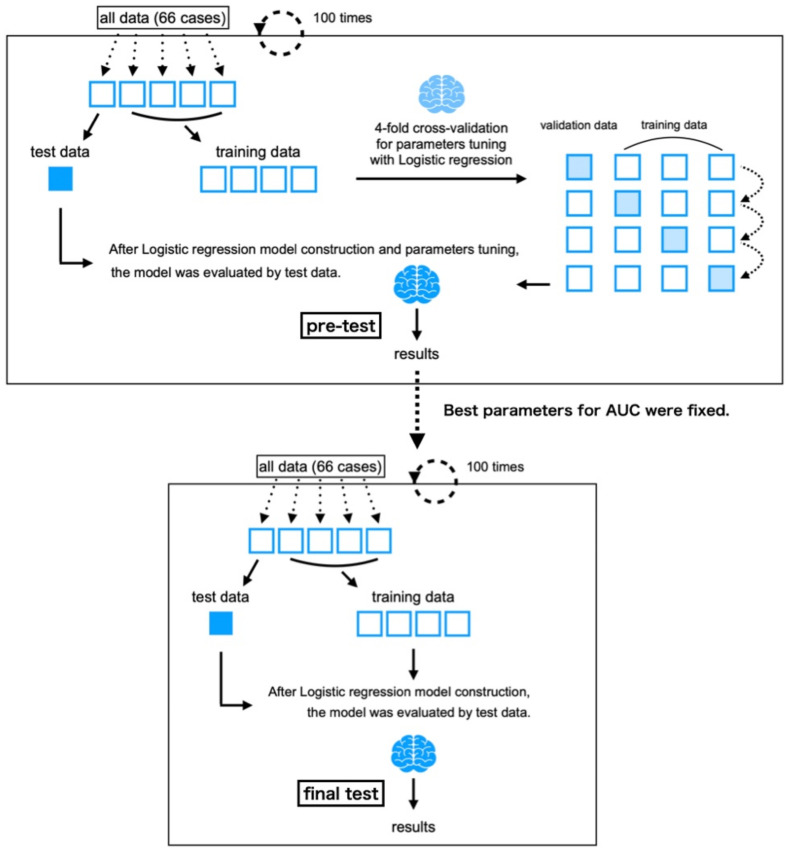
Algorithm shown in the box is a method of adjusting parameters to maximize the area under the curve (AUC). Then, as shown in the algorithm in the lower box, a logistic regression model was created with the training data by fixing the parameters at the most frequent values among those explored in the upper box, and the model was then evaluated with the test data.

We performed a two-stage experiment (Figure 3). The purpose of the first stage was to determine the hyperparameters. The second stage was to evaluate the classification performance and feature contributions.

We used logistic regression as the classification algorithm [19]. The loss function is the negative log-likelihood that is expressed by the following equation:

(1) $$E_{\left(\alpha \right)}=-\frac{1}{m}\overset{m}{\underset{i=1}{\sum }}\left\{w_{0}y_{i}\log\sigma_{\left(\alpha ,\;x_{i}\right)}+w_{1}\left(1-y_{i}\right)\log\left(1-\sigma_{\left(\alpha ,\;x_{i}\right)}\right)+\frac{1}{C}\overset{n}{\underset{j=1}{\sum }}{\left|\alpha_{j}\right|}^{2}\right\}$$

where m: number of training data, n, number of features; $x_{i,}$ sample I ${\left[x_{1},\;x_{2},\;x_{3},\cdots ,\;x_{n}\right]}^{T}$, $y_{i}:$ correct answer label of sample *i*, α: coefficients ${\left[\alpha_{1},\;\alpha_{2},\;\alpha_{3},\cdots ,\;\alpha_{n}\right]}^{T}$, C: regularization parameter, $\sigma_{\left(\alpha ,\;x\right)}:$ sigmoid function $1/\left(1+exp\left(-\alpha^{T}x\right)\right)$, $w_{0}:$ class weight of Group A, $w_{1}:$ class weight of Group B.

The above loss function $E_{\left(\alpha \right)}$ was minimized, and the coefficient α was updated with the limited-memory Broyden Fletcher Goldfarb Shanno (L-BFGS) algorithm. In the first stage of the experiment, stratified cross-validation was carried out as follows. First, we selected 20% of patients (i) as the testing data and the rest (ii) as the training data within the constraints that the ratio of Group A (*n* = 23) to B (*n* = 43) would be the same for (i) and (ii). Next, we carried out a stratified fourfold cross-validation for model construction and parameter tuning using (ii). To adjust the hyperparameters of the logistic regression, regularization parameter C was grid-searched at 0.001, 0.01, 0.1, 1, 10, and 100. After the model construction and parameter tuning, we evaluated the model using (i). The evaluation index in the grid search and the test is the area under the curve (AUC).

We conducted the above process 100 times for the determination of the hyperparameters. The most selected value of C in the first stage was adopted for the second stage to evaluate the classification performance and feature contributions.

In the second stage of the experiment, we conducted the same process as that in the first stage, but the grid search with stratified fourfold cross-validation was omitted because the regularization parameter C was determined. This is why the series of flow (data split, training, and tests) was carried out 100 times. Lastly, the classification performance and feature contributions were evaluated with the interval estimation of AUC and coefficients α. In addition, the class weight of Group A was applied 43/23 times more than the one of Group B because of the imbalanced data. The features were standardized before the machine-learning process. These tasks were implemented using the Python library scikit-learn [20].

## 3. Results

### 3.1. Demographics of Each Group

Some of the findings of this study were presented in our earlier paper [16]. In brief, 66 eyes of 66 patients who had met the eligibility criteria were studied, and the results of grouping by the visual acuity over time placed 23 eyes in Group A, and 43 eyes in Group B. The demographics of each group are listed in Table 1.

### 3.2. Explanatory Variables

Significant differences were observed in age, BCVA at the baseline, and BCVA at the time of the first resolution of the ME between Groups A and B. The results of the handcrafted features obtained from the OCT images at the first resolution of the ME are shown in Table 2. Significant differences were observed in the continuity of the ELM, continuity of the EZ, ELM-clarity, EZ-continuity-org, EZ-clarity, and area of the photoreceptors between Groups A and B. The R2 values were calculated to exclude variables with multicollinearity (Table 3). There were no multicollinearities between the variables that were statistically different between Groups A and B, and all were selected as explanatory variables.

### 3.3. Hyperparameters of Logistic Regression

**Figure 4:**
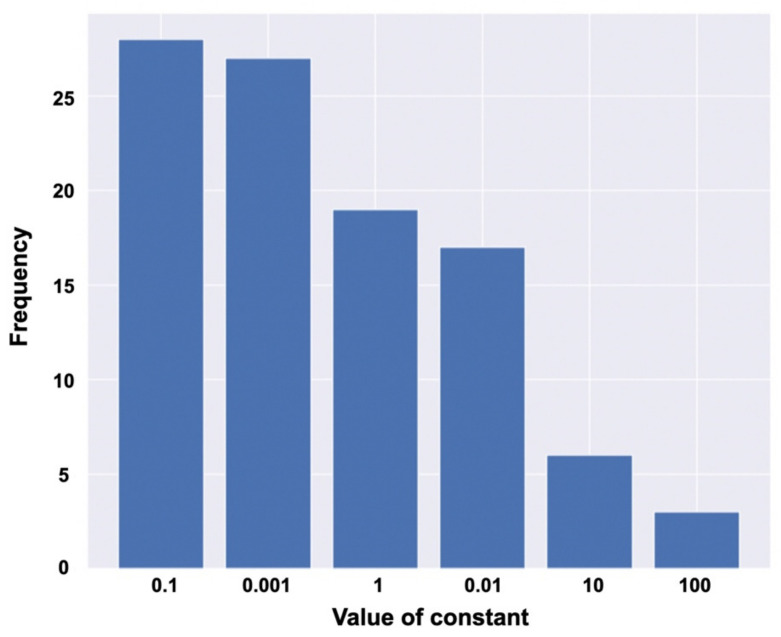
Frequency of each of the constant terms in the parameters.

The frequency of the occurrence of hyperparameters in 100 pretests for the adjustment of hyperparameters to maximize AUC is shown in Figure 4. On this basis, the hyperparameter used in the final test was fixed with a constant of 0.1.

### 3.4. Classification Performance

**Figure 5:**
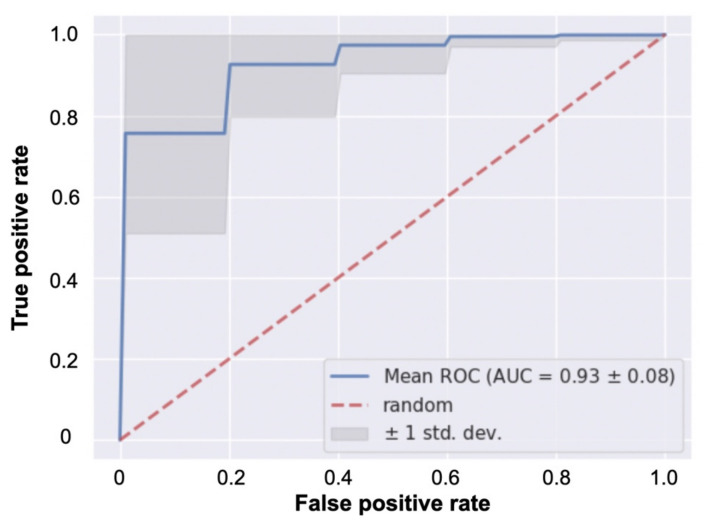
Mean receiver operating characteristic (ROC) curve of the classifier for Groups A and B with good and poor visual acuity over time during maintenance treatment. The standard deviation (SD) was equivalent to the SD of the area under the ROC curve (AUC) obtained by evaluating the model 100 times with the test data.

The predictive performance of classification between Groups A and B is shown in Figure 5. The mean AUC was 0.93, and the standard deviation was 0.08.

### 3.5. Specific Contribution of Explanatory Variables

**Figure 6:**
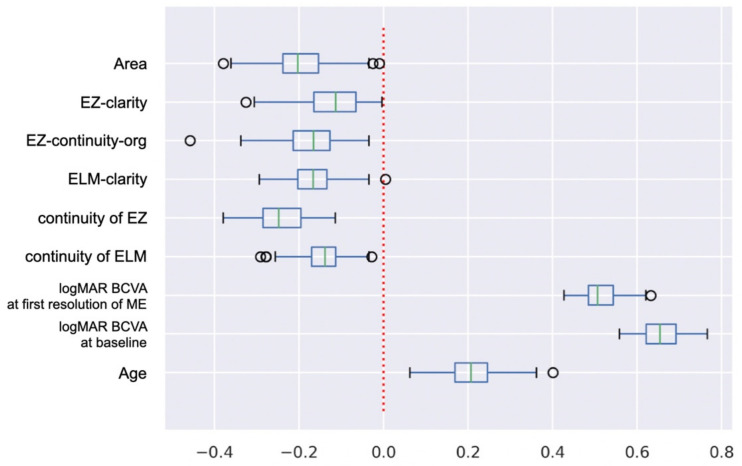
Box and whisker diagrams with outliers showing coefficients of explanatory variables. Maximal and minimal whisker lengths are set to the upper and lower limits of 1.5 times the interquartile range (IQR). First quartile −1.5 × IQR is the lower limit of the whisker, and third quartile + 1.5 × IQR is the upper limit of the whisker. Values smaller than the lower end of the whisker or larger than the upper end of the whisker are indicated by circles as outliers.

The determined regression coefficients are shown in Table 4 and Figure 6. The regression coefficients of the explanatory variables were: BCVA at baseline was 0.653, BCVA at first resolution of ME was 0.513, age was 0.210, EZ-clarity was −0.119, continuity of ELM was −0.144, ELM-clarity was −0.170, EZ-continuity-org was –0.173, Area was −0.198, and continuity of EZ was −0.244.

## 4. Discussion

In an earlier study, we used the same dataset with monthly visual acuity data for one year after the initial treatment as used in this study to classify two different prognoses of BCVA over time in eyes with ME due to a BRVO. The eyes were treated with the PRN regimen after the initial anti-VEGF injection. As shown in Table 1, our patients improved statistically significantly at 12 months post-treatment compared to the baseline logMAR BCVA. During the first year after the initial treatment of ME eyes with BRVO, BCVA fluctuated widely due to the high incidence of ME recurrence. Because BCVA at a given point in time does not always reflect the patient’s prognosis for visual function, we defined a threshold value on the basis of BCVA trends over time, and divided patients into two groups with different prognoses. The difference between the two studies was that, in the previous study, we used a nonlinear algorithm and support vector machine, but in this study, we used a linear algorithm and logistic regression. To the best of our knowledge, there have not been reports predicting the BCVA over time in eyes with ME due to BRVO from information at the time of first resolution of the macular edema after the initial anti-VEGF treatment. In that study, we used a nonlinear algorithm because we assumed that this classification task had an inherent complex nonlinear structure in it. The results showed that the classification performance was 0.806 for accuracy, 0.768 for precision, 0.772 for recall, and 0.752 for the *F*-measure. However, from the results of the current study, we found that the average accuracy of the linear algorithm was 0.93 AUC (Figure 4), showing that the linear algorithm was also capable of performing this classification task with high accuracy.

In constructing the prediction model, it was important to be able to understand the reason and accuracy of the model. The variables that were significantly correlated with the BCVA of eyes with ME associated with a BRVO were reported [4,5,6,7,9,10,21,22,23]. However, the causal relationships were too complex for clinicians to make prognostic predictions for their patients. Gallardo and colleagues used machine learning, and they also reported that the number of treatments could be predicted by extracting the variables from the OCT images [6]. Their study also obtained the degree of explanatory variables that contributed to the results. In our current study, the regression coefficients of the explanatory variables whose higher values predicted a poorer prognosis were, in order of influence, the BCVA at the baseline, the BCVA at the first resolution of the ME, and age (Table 4 and Figure 5). The BCVA at the baseline had the greatest influence on the prediction. There are reports on the post-treatment visual acuity, and its relationship to the pre- and post-treatment visual acuity after continuous anti-VEGF treatments [5,9,10]. This suggests the importance of starting treatments before the pretreatment BCVA becomes severe because the pretreatment BCVA worsens the later treatment is begun.

In terms of the BCVA at the time of the first resolution of the ME, the BCVA on the first day after the initial treatment was correlated with the BCVA at 6 months [23]. Although not as good as the pretreatment visual acuity, it was significantly correlated with the BCVA at the first resolution of the ME. The age was expected to have some influence on the prediction given the prevalence of atherosclerosis, in the elderly and the fact that vascular occlusion and stenosis are pathological conditions associated with BRVO [10]. Results show that the coefficients of age, and the means and standard deviations of the area of the outer segments of the photoreceptors were similar significant factors in predicting BCVA over time. However, age had a greater impact on the prediction than that of the findings of the OCT images.

On the other hand, the regression coefficients of the explanatory variables whose higher values predicted better prognosis were, in order of influence, the continuity of EZ, the area of the outer segments of the photoreceptors, EZ-continuity-org (brightness values of EZ), ELM-clarity (brightness values of ELM averaged in the vertical direction), continuity of ELM, EZ-clarity (brightness values of EZ averaged in the vertical direction), continuity of ELM, and the area of the outer segments of the photoreceptors. For this classifier, information of the demographics of the patients was needed more than that of the images. However, information obtained from the OCT images was also important for the predictions.

There are several reports on the relationship among OCT morphological findings, such as the EZ [13,14,24], area of the outer segments of the photoreceptors [11,12], and the integrity of the ELM and the visual acuity in eyes with BRVO [14]. We determined the variables for our study on the basis of these findings. Looking at the coefficients of the above six variables, the top three were the continuity of the EZ, the area of the outer segments of the photoreceptors, and the EZ-continuity-org, the brightness values of EZ. These variables were derived from the photoreceptors, and the results suggest that maintaining the normality of these structures may be a goal of the initial treatment. The ELM-clarity, i.e., brightness values of ELM averaged in vertical direction, was the fourth highest coefficient. As Hasegawa and colleagues inferred that the ELM is a structure that stops photoreceptor damage caused by intraretinal fluid [14], the present results suggest that the ELM may play an important role in visual function during the maintenance treatment.

The OCT B-scan images were used when the macular edema had disappeared, and the image was cropped to 2 mm. There were two reasons for this cropping. First, the images had the least variability in the induction phase of the treatment. Because our dataset was small, it was important to minimize the variations. The second reason for cropping was because the cell density of the macula is exponentially higher from the periphery to the center [25]. Within 2 degrees of the center of the macula, the density of cone cells is especially high [25,26], which is the region that contributes significantly to BCVA [27]. Curcio and colleagues reported that there are 50,000 cones/mm^2^ or more within 2 degrees of the center of the macula [25], and Woog and colleagues reported that the visual function within 2 degrees of the center of the macula corresponded to 0 logMAR units or better [27]. We annotated the OCT image within 2 degrees of the central macular to define the continuity and brightness values of the ELM and EZ, and the area of the outer segments of the photoreceptors as handcrafted features.

There are limitations in this study. One limitation was the small sample size of 66 eyes. Even though it would have rendered the classifier more suitable, it would need a longer time to collect a greater amount of clean data of BRVO treated cases that met the eligibility criteria. The second limitation regarded the selection of the explanatory variables. As in our previous report, we used a filter method, and variables that showed significant differences with the targeted variable were used as the explanatory variables [16]. This is why explanatory-variable selection is the simplest and easiest way to determine whether the explanatory variable was adopted or not. However, the limitation of this method is that we could only consider the variables that we had selected. If we could use deep learning to extract the explanatory variables from the image itself, letting the algorithm perform the work, we could overcome this limitation. However, due to the limited number of samples, deep learning could not be used. Lastly, the contribution of the coefficients of these variables only shows the trend of the prediction model in this dataset and not the result of generalizing the contribution of the variables of the cause of good or poor prognosis.

## 5. Conclusions

In conclusion, a classifier was created with handcrafted-feature-based logistic regression that adjusted the parameters to maximize the AUC with accuracy of 0.93. This algorithm predicted the visual function prognosis over time for individual patients with ME associated with BRVO before continuous anti-VEGF monotherapy. The patients’ clinical information and OCT findings at the first resolution of the ME was helpful in the classification of the different prognoses of the BCVA during continued anti-VEGF treatment. The contribution of the explanatory variables to the classifier was also found.

## Figures and Tables

**Table 1:** Baseline patient characteristics.

Variables	All (*n* = 66)	Group A (*n* = 23)	Group B (*n* = 43)	*p* Value
Men, *n* (%)	36 (54.5)	17 (73.9)	19 (44.2)	0.368
Age (y), mean ± SD	67.2 ± 9.4	63.6 ± 12.1	69.2 ± 6.9	0.024 *
Duration from onset to treatment (weeks), mean ± SD	6.86 ± 7.05	4.96 ± 3.34	7.90 ± 8.28	0.147
Type, Major/macula (%)	44(66.7)/22(33.3)	15(22.7)/8(12.1)	29(44.0)/14(21.2)	1
Affected eye, right/left (%)	35(53.0)/31(47.0)	10(15.2)/13(19.7)	25(37.9)/18(27.3)	0.299
Location of affected semifield, superior/inferior (%)	48(72.7)/18(27.3)	17(25.8)/6(9.1)	31(47.0)/12(18.2)	1
logMAR BCVA at baseline, mean ± SD	0.41 ± 0.30	0.15 ± 0.15	0.54 ± 0.28	<0.001 *
logMAR BCVA at first resolution of ME, mean ± SD	0.22 ± 0.24	0.04 ± 0.09	0.32 ± 0.24	<0.001 *
Period from initial treatment to the first resolution of ME (weeks), mean ± SD	4.5 ± 1.6	4.5 ± 1.4	4.5 ± 1.7	0.147
logMAR BCVA at 12 months, mean ± SD	0.01 ± 0.16	−0.01 ± 0.06	0.16 ± 0.17	<0.001 **
Number of anti-VEGF therapy at 12 months	3.2 ± 1.9	2.6 ± 2.0	3.5 ± 1.9	0.26

SD = standard deviation; logMAR = logarithm of minimal angle of resolution; BCVA = best-corrected visual acuity; *p* values were calculated by comparing each variable between Groups A and B. Variables marked with * are the results of tests of comparisons between Groups A and B, which were considered significant at *p* < 0.05 (Kolmogorov–Smirnov test was used for continuous quantitative variables. Fisher’s exact test was used for categorical variables). Variables marked with ** are those that were considered significant at *p* < 0.05 in the test of comparison with logMAR BCVA at baseline for all eyes.

**Table 2:** Features of trimming image at the first resolution of ME.

Variables	All (*n* = 66)	Group A (*n* = 23)	Group B (*n* = 43)	*p* Value
Continuity of ELM, defect/discontinuous/continuous (%)	6(9.1)/12(18.2)/48(72.7)	0/1(1.5)/22(33.3)	6(9.1)/11(16.7)/26(39.4)	0.007 *
Continuity of EZ, defect/discontinuous/continuous (%)	8(12.1)/26(39.4)/32(48.5)	0/5(7.6)/18(27.3)	8(12.1)/21(31.8)/14(21.2)	<0.001 *
ELM-continuity-org	5021 ± 1271	5019 ± 1358	5021 ± 1271	0.295
ELM-clarity	1.14 ± 0.12	1.20 ± 0.10	1.10 ± 0.12	0.001 *
EZ-continuity-org	6911 ± 2352	8140 ± 1827	6239 ± 2353	0.006 *
EZ-clarity	1.14 ± 0.12	1.21 ± 0.11	1.11 ± 0.12	<0.001 *
Area	615 ± 203	689 ± 124	576 ± 228	0.006 *

ELM = external limiting membrane; EZ = ellipsoid zone; continuity-org = total sum of the brightness values under the ELM or EZ line; clarity = total sum of the ratio between the brightness value below the ELM or EZ line and the average value of the brightness values of 3 pixels above and below it; Area = area surrounded by the EZ and RPE lines; *p* values were calculated by comparing each variable between Groups A and B. *, significant at *p* < 0.05 (Kolmogorov-Smirnov test was used for the continuous quantity variables. Fisher’s exact test was used as the categorical variables).

**Table 3:** Evaluation of multicollinearity.

Variables	R^2^ Score	Judge
Age	0.115	False
logMAR BCVA at baseline	0.382	False
logMAR BCVA at first resolution of ME	0.613	False
Continuity of ELM	0.623	False
Continuity of EZ	0.638	False
ELM-clarity	0.655	False
EZ-continuity-org	0.416	False
EZ-clarity	0.595	False
Area	0.362	False

logMAR = logarithm of minimal angle of resolution; BCVA = best-corrected visual acuity; ELM = external limiting membrane; EZ = ellipsoid zone; continuity-org = total sum of the brightness values under the ELM or EZ line; clarity = total sum of the ratio between the brightness value below the ELM or EZ line and the average value of the brightness values of 3 pixels above and below it; Area = area surrounded by the EZ and RPE lines.

**Table 4:** Coefficients of explanatory variables.

Variables	Mean	SD	95% Confidence Interval of the Mean	
			Lower Limit	Upper Limit
logMAR BCVA at baseline	0.656	0.049	0.646	0.666
logMAR BCVA at first resolution of ME	0.513	0.039	0.505	0.521
Age	0.210	0.070	0.196	0.224
EZ-clarity	−0.119	0.069	−0.133	−0.105
Continuity of ELM	−0.144	0.050	−0.154	−0.134
ELM-clarity	−0.170	0.062	−0.182	−0.158
EZ-continuity-org	−0.173	0.075	−0.188	−0.158
Area	−0.198	0.076	−0.213	−0.183
Continuity of EZ	−0.244	0.059	−0.255	−0.232

The greater the value of the explanatory variable with a positive coefficient, the more likely it was to be classified in Group B. On the other hand, the greater the value of the explanatory variable with a negative coefficient was, the more likely it was to be classified in Group A. SD = standard deviation; logMAR = logarithm of minimal angle of resolution; BCVA = best-corrected visual acuity; ELM = external limiting membrane; EZ = ellipsoid zone; continuity-org = total sum of the brightness values under the ELM or EZ line; clarity = total sum of the ratio between the brightness value below the ELM or EZ line and the average value of the brightness values of 3 pixels above and below it; Area = area surrounded by the EZ and RPE lines.

## Data Availability

All data included in this study are available from the corresponding author upon reasonable request.
